# Editorial: Medical Education in Difficult Circumstances

**DOI:** 10.15694/mep.2017.000001

**Published:** 2017-01-09

**Authors:** Trevor Gibbs, Michelle McLean, Ewa Pawlowicz, Judy McKimm

**Affiliations:** 1 AMEE; 2 Bond University Medical School; 3 Medical University of Lodz; 4 Swansea University Medical School

**Keywords:** Medical and healthcare education, overcoming difficulties, problems and solutions

## Abstract

This article was migrated. The article was marked as recommended.

Not indicated.

## Introduction

***“Let us pick up our books and our pens. They are our most powerful weapons. One child, one teacher, one book and one pen can change the world.”*** Malala Yousafzai (United Nations General Assembly, 2013)

These are the words of a brave 15-year-old girl who had recovered after being shot in the head the year before for courageously taking a stand against an oppressive regime that opposed education for women. Against the odds, she fought for a cause in which she believed. For Malala, education provides each of us (as well as collectively) with the power to change the *status quo.* Medical and health professional education has the power to change lives, but for many, challenges and difficulties need to be overcome.

We live in an increasingly volatile, uncertain, complex and ambiguous (VUCA) world, a world that is plagued by war, conflict, political upheaval, emerging epidemics and natural disasters ( Lemoine et al., 2017). Events of the last few years, particularly in the Middle Eastern region, has led to millions of refugees, including some health care practitioners and students, who have left their country of birth in search of a safer place to hopefully study or work. Amidst the challenges of this complex world, medical education, like life, must continue but many students and doctors have to study and work in the most difficult of circumstances, perhaps even under duress. The description above reflects some of the most extreme difficult circumstances, lying at the furthest end of a spectrum, which ranges from terrible and tragic situations to more mundane or entrenched, but important issues, such as lack of teaching resources, institutional sexism or racism, rigidity in curriculum development and financial constraints.

In 2007, Gibbs wrote that: “ *As an international community that is seemingly passionate about medical education and shares a belief that the future of a country’s health relies on the education of its future healthcare workers we* (as an international community of educators *) have to sit up and recognise the problems faced by our peers*”. Almost a decade later, as medical educators, we still have to ask ourselves whether we are indeed doing our best to assist our colleagues and their students who may be experiencing a range of difficulties and challenges studying or working.

AMEE, the Association for Medical Education in Europe, has taken a stand. Recognising that ‘difficulties’ are contextual and, to better understand the spectrum of challenges facing medical educators across the globe (with the view to being able to offer possible strategies or solutions in some circumstances), *‘Medical Education in Difficult Circumstances’* was identified as a theme for the 2016 AMEE Conference in Barcelona. Based on the information gathered at the various plenaries, symposia, oral and poster presentations and a specific workshop, this editorial attempts to provide some insight into the deliberations of the conference participants and provides the stimulus for AMEE members to contribute to the *MedEdPublish* January-March 2017 themed issue of *‘Medical Education in Difficult Circumstances’.*

First, we offer a description of what ‘difficult circumstances’ means, a collection of meanings which emerged from the 2016 AMEE Conference workshop:

A ‘difficult circumstance’

• Is recognised as being out of the ordinary, i.e. beyond what is difficult in everyday life (and which may be context-specific);
• May result from a conflict of values or beliefs;
• Can impact at different levels, ranging from an individual, an institution or organisation or even a system (e.g. health care system);
• Can be an acute or crisis situation or a long term issue;
• Does not allow goals to be achieved;
• May be morally distressing;
• Can impact on the mental and physical well-being of students or faculty.

At one of the 2016 AMEE plenaries, Dr Ewa Pawlowicz, a recently graduated doctor from Poland, presented a student perspective on *‘Medical Education in Difficult Circumstances’.* Ewa’s summary below reminds us to work with students as partners as we deliberate on the many challenges we collectively face in medical education:

*“Students, if treated as partners in addressing and responding to challenges, are of a great value to medical education. Since students ‘suffer’ directly from a range of ‘difficulties’ in their medical training and education, they should become active agents in helping to resolve some of the problems. As students ‘live’ the curriculum, they can often identify issues long before they become difficulties. With their fresh approach to ‘seeing’ things, students can be agents of change, which is particularly important in countries where curriculum models are out-dated, traditional and overloaded with theoretical knowledge.*

*International student organisations such as the International Federation of Medical Students’ Association (IFMSA) or the European Medical Students’ Association (EMSA), provide students with several opportunities for sharing experience and knowledge about different health care and medical education systems as well as facilitating their participation in exchange programmes. Although internships abroad should be an integral part of curricula, many medical students are still denied the opportunity of taking part in them.*

*Students should constantly develop their knowledge and awareness about medical education, what would allow them to become reliable and proper partners for medical school authorities. Raising students’ responsibility for their learning and education is probably the best way to increase their motivation to engagement. Students’ organisations should also create long-term strategies and consolidate their actions, what might lead to better recognition of students’ voice in the academic community. Thanks to these actions students may become true change leaders in medical education“.*

The authors would value additional perspectives from students and doctors in training in terms of the difficulties they have or face and how they cope.

During the same plenary, Professor Philip Cotton, Vice-Chancellor of the University of Rwanda Medical School, provided an insightful account of how the restructuring of a University and its medical school, in the aftermath of one of the bloodiest conflicts seen in Africa, has been achieved through a common vision and a determination to succeed. Below is a summary from Professor Cotton about his unifying work at the University of Rwanda.

“ ***Stumbling blocks into stepping stones; celebrating medical education in Rwanda.***

*The University of Rwanda was created two years ago (2014) from the merger of the seven public Universities. There are now 31,000 students on 14 campuses. It is the majority provider of doctors and nurses, and the sole provider of all other health care professionals. The merger is complete and during these two years, in response to predicted needs, we have opened the first-ever dental school, doubled the intake into medicine, and started the first ever Masters degrees in clinical nursing for 160 candidates. The challenges facing students and faculty, and delivery of teaching in clinical environments, are not new but the opportunities that emerge are exciting and energising.*”

In his plenary, Professor Cotton highlighted that the ability to move forward in difficult circumstances requires, amongst many other things, high-level administrative support. We therefore invite university and faculty administrators, programme managers and curriculum support staff to share their experiences by contributing to this themed issue of MedEdPublish.

Table 1 provides a summary of a number of the identified challenges and difficulties that emerged from the fruitful discussions during the AMEE workshop, “Medical Education in Difficult Circumstances”, in Barcelona. Also provided are some examples and contexts as well as some broad suggestions and strategies for coping and dealing with the identified issues. We believe, however, that there is much work that still needs to be done and therefore appeal to the international community of health professions’ educators and students to assist in identifying additional ‘difficult circumstances’ or offer strategies and solutions to those which have already been identified.

## Conclusions

With events such as the Paris Agreement on reducing carbon emissions to mitigate climate change now international law and the recent election of Donald Trump as the next US president, we move into a new era in our global history. Some may claim that our future as a global community is uncertain. What we do know, however, is that change will happen. We also know that with change come challenges, some of which may be painful. As a global community of medical and health professional educators, we stand at a crossroads. We have two options: we can either bury our heads in the sand (for which we may pay dearly later) or we can step up and work collaboratively to tackle these difficulties and support our colleagues. We believe we need to take the latter path. We hope that by identifying common difficulties in medical and health professions education, we can collectively find solutions and strategies to overcome some of the most pressing issues facing students and doctors today and tomorrow.

## Notes On Contributors

**Professor Trevor Gibbs, MD, SFHEA, DA. FAcadMED, MMedSc, FRCGP, FAMEE** - Trevor is an independent Professor and Consultant in Medical Education and Primary Care. As Deputy Editor of Medical Teacher he has specific responsibility for the development of AMEE Guides, the BEME Guides, and the Medical Education around the World series. His experience in General Practice and interest in medical education have given him the opportunity to develop curricula in many parts of the world, specifically in those regions in which medical and healthcare education is often a challenge. He has a special interest in the social accountability of medical schools.

**Prof Michelle McLean, MSc, PhD, MEd** - Professor Michelle McLean is the Academic Lead for Problem-based Learning (PBL) in the undergraduate medical programme at Bond University, Gold Coast, Australia. Having worked on three continents in three very different contexts, Michelle’s interests include diversity in learning and teaching and preparing the future medical workforce for an increasingly complex world. Her interest in ‘Medical Education in Difficult Circumstances’ stems from growing up in South Africa during Apartheid and being in an academic position when transformation of higher education was required to address decades of inequity.

**Ewa Pawłowicz** graduated from medical faculty of Medical University of Lodz in 2015, and is at present studying for a clinical PhD. As a student she was an member of International Federation of Medical Students’ Associations (IFMSA) and worked for Standing Committee on Medical Education, taking part in many international meeting and projects. She participated in clinical exchange programmes in Sweden, USA and Portugal. Since 2013 she has cooperated with Centre for Medical Education in Lodz with a focus on enhancement of student engagement in medical education in Poland.

**Professor Judy McKimm, MBA, MA(Ed), BA(Hons), PGDip (HSW), SFHEA, FAcadMed’s** - Judy’s current role is Director of Strategic Educational Development and Professor of Medical Education in the College of Medicine, Swansea University. Judy initially trained as a nurse and has an academic background in social and health sciences, education and management. She is programme director for the Leadership Masters at Swansea and Director of ASME’s International Educational Leadership programme.

## Declarations

Prof Trevor Gibbs, Prof Judy McKimm and Prof Michelle McLean are all guest Theme Editors for the AMEE MedEdPublish themed issue for Medical Education in Difficult Circumstances.

## Appendices

**Table 1.** Categories of difficulties identified in medical and health professions’ education.

Issues identified	Identified circumstance	Examples	Strategies and solutions
**Global**	**Unsafe, dangerous situations** • Natural disasters; climate change • War and conflict • Epidemics and outbreaks (e.g. Ebola, foot and mouth) **Political/economic issues** • Neoliberalism (commodification) • Populism	• Earthquakes, floods, fires • Shortages of doctors and other HPs • Doctors being killed in conflict • Difficulty in recruitment (also a general issue) • Accreditation standards, lack of regulation • Expenditure cuts	No specific solutions were provided for any of these issues. Suggestions were made mainly about raising awareness and coping strategies. **Broad strategies:** • Postpone or abandon the programme • Deal with the issue in the short- or long-term - adaptation, collaboration • Mitigate damage, normalise the situation
**Health and education systems**	• Low resource or remote settings • University funding models • Competing values; role conflict • Quality of education • Multiculturalism	• Difficult to recruit doctors: Leads to a low doctor: patient ratio, e.g. In the Congo, 77 ophthalmologists for 80 million people so preventable diseases (e.g. cataract) prevail • Also affects clinical supervision • CPD and faculty development not offered • Declining health care systems • Fees (e.g. student unrest in South Africa) • Country/regional needs vs. individual needs • Education vs. service delivery • Education vs. research • Private vs. public education • Low staff: student ratio • Traditional paradigm; resistance to change • Accreditation standards, lack of a national exam • Non-inclusive curriculum • Conflict of beliefs and values • Inequality	**Suggestions:** • Use of telemedicine • Students need to undertake volunteer work to develop a sense of patient advocacy **General suggestions (for a range of circumstances):** • Just doing something (action) • Plan (reflection)- implement- evaluate-reflect (Kolb’s experiential learning cycle) • Make slow but sure changes • Celebrate small successes • Change the system or develop flexible systems • Plan for the long-term (requires patience) • Develop a sense of ownership (through good leaders) • Identify champions • Positive role models • Collaborate and share good practice with like-minded individuals • Transcultural competence training • Use narratives, stories and conversations • ‘Fit for purpose’ curriculum
**Organisational; teamwork**	Medical hierarchy; lack of group or institutional cohesion	• Conflict amongst health professionals • Working with individual purpose and/or power • Gender, race, religious, etc. discrimination	• Interprofessional training • Unconscious bias training • Institutional sense of purpose • Clear, effective leadership
**Individual or personal**	Uncertainty	Lack of student support systems	• Development and empowerment of the student body • Develop resilience by exposing students to change
