# Supplementary material for: Editorial: Medical Education in Difficult Circumstances
Source: MedEdPublish (2016). 2017 Jan 9;6:1. [Version 1] doi: 10.15694/mep.2017.000001 (PMC10702861; doi:10.15694/mep.2017.000001)
Supplement: Peer review history

# Reviewer report: Reviewer response for version 1

**DOI:** 10.21956/mep.19411.r28490

**Published:** 2018-04-13

**Authors:** Davinder Sandhu

**Affiliations:** 1 Royal College of Surgeons in Ireland, Medical University of Bahrain, POBox 15503, Adliya, Bahrain.

This review has been migrated. The reviewer awarded 5 stars out of 5 Excellent and moving piece and it is pleasing to see that the insightful session at Barcelona has not been lost and the main messages captured. This article is of global significance which probably sounds dramatic but the 50% who are the haves and the other 50% who are the have nots all all included. One cannot work without the other. Well done Gibbs et al and AMEE.

Reviewer Expertise:

NA

No decision status is available

# Reviewer report: Reviewer response for version 1

**DOI:** 10.21956/mep.19411.r28487

**Published:** 2017-01-20

**Authors:** Yingzi Huang

**Affiliations:** 1 The First Affiliated Hospital, Sun Yat-sen University

This review has been migrated. The reviewer awarded 5 stars out of 5 It’s an inspiring although saddening opening remark, given that it involves issues as difficult as wars and major disasters. I can’t agree more to quote Malala as a role model for medical education in difficult circumstances, who was also mentioned earlier by Professor Trevor Gibbs in his first ESME course for the First affiliated hospital, Sun Yat-sen Medical University. One may say though Malala’s just lucky to have survived after being shot. Still no one can deny that she deserves the Nobel Prize as a teenager, given the underlying positive psychology and stepping up to inequality, sending so much hope and faith in education to the rest of the world despite all the life-threatening difficulties. It’s an inspiring article for China as well, which reminds me that development of medical education was not smooth from the very beginning with stumbling blocks. Like invisible wars, bureaucracy and inefficiency are not uncommon within the system, where innovation and change won’t happen without resilience. Thanks to the leaders’ determination and collaboration between AMEE and China, many ‘first’ s for China and Sun Yat-sen arose and many more will follow as more practical solutions promoted by people who share the spirit of Malala, who keeps fighting for what we all believe. Thus I’m also excited to read Professor Xiao Haipeng’s review which addresses quite a few of his thoughts for the future, faculty development being one of the highlights certainly.One of my classmates in CUHK is a Yemeni lady, whose research is about role of public health in war, the norm in her country. The same powerful weapons chosen by more, Malala is NOT alone.

Reviewer Expertise:

NA

No decision status is available

# Reviewer report: Reviewer response for version 1

**DOI:** 10.21956/mep.19411.r28488

**Published:** 2017-01-13

**Authors:** Haipeng Xiao

**Affiliations:** 1 The First Affiliated Hospital, Sun Yat-sen University

This review has been migrated. The reviewer awarded 5 stars out of 5 This is a well-written editorial that provides an overview of contextualized difficulties in medical education throughout the world, pointing out underlying ones as well for further solutions. Crucial as the issue is, we rarely find any relevant article about how to be the voice of the less fortunate and help tackle the difficulties in a timely and effective manner. This inspiring article is thus especially helpful for those involved in difficult circumstances. Table 1 is a very useful ‘tool’ of analysis of uncommon situations for finding possible way out. Build coping mechanism and promoting positive psychology are also highlighted, which are tremendously important in time of difficulties.There are probably still some issues worth addressing: 1. In table 1, considering the severity of the issues, broad strategies for Global issues could be more: a. promote hope and resilience by relevant NGO, Government’s social accountability; b. Demonstration of immediate awareness and prompt competence,c. Humanitarian aids and civilian protection; d. Post-conflict reconstruction; e. Local empowerment and capacity building.2. In the health and education system issue, examples for low resources may include lack of quality supervision of education, lack of feedback, lack of motivation mechanism. Examples for competing values could involve student-centered vs teacher-centered. Solution could be establishment of motivation mechanism. 3. In the organizational teamwork issue, the examples could be “bureaucracy and inefficiency”. Solution could be ’ promotion of evidence-based medical education’. 4. The examples for “individual or personal issues” could be “lack of faculty support system” , and the solution could be ‘faculty development’.

Reviewer Expertise:

NA

No decision status is available

# Reviewer report: Reviewer response for version 1

**DOI:** 10.21956/mep.19411.r28489

**Published:** 2017-01-11

**Authors:** Richard Hays

**Affiliations:** 1 James Cook University

This review has been migrated. The reviewer awarded 5 stars out of 5 Thanks to the authors for this summary of some of the challenges that face medical educators and health professional learners throughout the world. Medical education can be complex anywhere, but in much of the developed world there is relatively secure funding, plenty of high quality candidates for faculty positions, political stability and personal safety. The session at the recent AMEE meeting highlighted the differences that many of our colleagues face in trying to do the same job as those of us in more fortunate circumstances. It will be interesting to see where this discussion leads. At the moment the title of the theme is probably deliberately broad. However, this means that a very wide range of complex issues are included, and makes difficult the task of working on solutions, which may also be complex. I look forward to reading other contributions to this themed issue, as through open discussion it may be possible to develop practical advice and assistance to overcome some of the difficult circumstances. After all, in the increasingly globalised world, we all have a vested interest in seeing high standards of medical education and medical practice everywhere.

Reviewer Expertise:

NA

No decision status is available
